# Third vaccine boosters and anti‐S‐IgG levels: A comparison of homologous and heterologous responses and poor immunogenicity in hepatocellular carcinoma

**DOI:** 10.1002/kjm2.12812

**Published:** 2024-02-16

**Authors:** Chih‐Wen Wang, Chung‐Feng Huang, Tyng‐Yuan Jang, Ming‐Lun Yeh, Po‐Cheng Liang, Yu‐Ju Wei, Po‐Yao Hsu, Ching‐I. Huang, Ming‐Yen Hsieh, Yi‐Hung Lin, Jee‐Fu Huang, Chia‐Yen Dai, Wan‐Long Chuang, Ming‐Lung Yu

**Affiliations:** ^1^ Division of Hepatobiliary, Department of Internal Medicine Kaohsiung Medical University Hospital Kaohsiung Taiwan; ^2^ School of Medicine and Hepatitis Research Center, College of Medicine and Center for Liquid Biopsy and Cohort Research Kaohsiung Medical University Kaohsiung Taiwan; ^3^ Department of Internal Medicine, Kaohsiung Municipal Siaogang Hospital Kaohsiung Medical University Kaohsiung Taiwan; ^4^ Ph.D. Program in Translational Medicine, College of Medicine Kaohsiung Medical University, Kaohsiung, and Academia Sinica Taipei Taiwan; ^5^ Faculty of Medicine Kaohsiung Medical University Kaohsiung Taiwan; ^6^ Department of Internal Medicine, Kaohsiung Municipal Ta‐Tung Hospital Kaohsiung Medical University Kaohsiung Taiwan; ^7^ Division of Hepatogastroenterology, Department of Internal Medicine Kaohsiung Chang Gung Memorial Hospital Kaohsiung Taiwan; ^8^ School of Medicine, College of Medicine and Center of Excellence for Metabolic Associated Fatty Liver Disease National Sun Yat‐Sen University Kaohsiung Taiwan

**Keywords:** AZD1222, BNT162b2, chronic liver disease, hepatocellular carcinoma, mRNA‐1273

## Abstract

The immune response of patients with chronic liver disease tends to be lower after receiving their second coronavirus disease 2019 (COVID‐19) vaccine dose, but the effect of a third vaccine dose on their immune response is currently unknown. We recruited 722 patients without previous severe acute respiratory syndrome coronavirus 2 (SARS‐CoV‐2) infection from three hospitals. The patients received homologous (MMM) and heterologous (AZAZBNT, AZAZM) boosters, where AZ, BNT, and M denoted the AZD1222, BNT162b2, and mRNA‐1273 vaccines, respectively. Serum IgG spike antibody levels were measured at a mean 1.5 ± 0.7 (visit 1) and 5.0 ± 0.5 (visit 2) months after the third vaccine booster. A threshold of 4160 AU/mL was considered significant antibody activity. In both visits, the patients who received the MMM booster had higher anti‐S‐IgG levels than those who received the AZAZBNT and AZAZM boosters. Patients with active hepatocellular carcinoma (HCC) had lower anti‐S‐IgG levels than the control group (761.6 vs. 1498.2 BAU/mL; *p* = 0.019) at visit 1. The anti‐S‐IgG levels decreased significantly at visit 2. The patients with significant antibody activity had a lower rate of liver cirrhosis with decompensation (0.7% decompensation vs. 8.0% non‐decompensation and 91.3% non‐liver cirrhosis, *p* = 0.015), and active HCC (1.5% active HCC vs. 3.7% non‐active HCC and 94.7% non‐HCC, *p* < 0.001). Receiving the MMM booster regimen (OR = 10.67, 95% CI 5.20–21.91, *p* < 0.001) increased the odds of having significant antibody activity compared with the AZAZBNT booster regimen. Patients with active HCC had a reduced immune response to the third COVID‐19 vaccine booster. These findings underscore the importance of booster vaccinations, especially in immunocompromised patients, with superior efficacy observed with the homologous mRNA‐1273 regimen.

**Abbreviations**
AASLD: American Association for the Study of Liver Diseases
anti‐S: anti‐spike protein
AZ: AZD1222 vaccine
BNT: BNT162b2 vaccine
CI: confidence interval
COVID‐19: coronavirus disease 2019
EASL: European Association for the Study of the Liver
HBV: hepatitis B virus
HCC: hepatocellular carcinoma
HCV: hepatitis C virus
M: mRNA‐1273 vaccine
NAFLD: non‐alcoholic fatty liver disease
OR: odds ratio

## 1 INTRODUCTION

Patients with chronic liver disease infected by severe acute respiratory syndrome coronavirus 2 (SARS‐CoV‐2) are at a significantly increased risk of mortality.^1^, ^2^, ^3^ In addition, a previous large cohort study reported that liver cirrhosis was significantly associated with an increased risk of mortality and hospital admission in individuals after one or two doses of the AZD1222 or BNT162b2 vaccine booster.^4^ Innate and adaptive immune responses to immunogenicity after SARS‐CoV‐2 vaccination in patients with liver cirrhosis are therefore a cause of great concern. A previous study of subjects receiving two doses of inactivated SARS‐CoV‐2 vaccine revealed that the seroconversion rates of neutralizing antibody were 90.3% in healthy subjects compared with 76.8%, 78.9%, and 76.7% among non‐cirrhosis, compensated and decompensated cirrhosis groups, respectively.^5^ In addition, a population‐based study reported that the antibody responses 40 days after two dose of SARS‐CoV‐2 vaccination were poor in 24% of patients with chronic liver disease and in 61% of those who underwent a liver transplantation.^6^ However, previous studies have demonstrated good immunogenicity after two doses of inactivated vaccine against SARS‐CoV‐2 in patients with non‐alcoholic fatty liver disease (NAFLD) and hepatitis B virus (HBV) infection.^7^, ^8^

The type of vaccine used has been shown to have an impact on immunogenicity.^9^ Previous studies have demonstrated that both homologous and heterologous booster vaccines (including mRNA‐1273, Ad26.COV2. S, and BNT162b2) are safe and immunogenic in healthy adults.^10^ Moreover, a study of adults aged 50 years and older with no or well‐controlled comorbidities who received two doses of vaccination showed greater increases in antibody levels after heterologous boosting than after homologous boosting.^11^ In addition, a retrospective cohort study of the Veterans database conducted by John et al. found that a third dose of a coronavirus disease 2019 (COVID‐19) mRNA vaccine was associated with significant reductions in COVID‐19 and its severity, including an 80.7% reduction in COVID‐19, an 80.4% reduction in symptomatic COVID‐19, an 80% reduction in moderate, severe, or critical COVID‐19, a 100% reduction in severe or critical COVID‐19 (95% confidence interval [CI] 99.2–100.0, *p* = 0.01), and a 100% reduction in COVID‐19‐related deaths in patients with liver cirrhosis.^12^

Furthermore, the European Association for the Study of the Liver (EASL) and the American Association for the Study of Liver Diseases (AASLD) both recommend that COVID‐19 vaccinations should be prioritized for patients with advanced liver disease, as well as those with immune‐mediated liver disease who are on immunosuppressive therapy.^13^, ^14^ However, studies examining the response to COVID‐19 vaccines in patients with chronic liver disease after a third vaccine dose are limited, and the impact of homologous and heterologous vaccine immunogenicity has not been investigated previously.^15^ Therefore, the objective of this study was to investigate the anti‐spike protein (anti‐S) IgG response in patients with HBV infection, hepatitis C virus (HCV) infection, NAFLD, liver cirrhosis, and hepatocellular carcinoma (HCC) following the administration of a third dose of either a homologous or heterologous COVID‐19 vaccine.

## 2 MATERIALS AND METHODS

### 2.1 Ethics statement

The study protocol was approved by the Institutional Review Board of Kaohsiung Medical University Hospital. Written informed consent was obtained from all participants, and all clinical investigations were conducted in accordance with the principles of the Declaration of Helsinki.

### 2.2 Participant recruitment

We conducted a prospective cohort study at Kaohsiung Medical University Hospital, Kaohsiung Municipal Siaogang Hospital, and Kaohsiung Municipal Ta‐Tung Hospital in southern Taiwan from 2021 to 2022. We consecutively recruited participants who had received third booster doses of BNT162b2 or mRNA‐1273. We recorded details of chronic liver diseases including chronic HBV infection, HCV infection, NAFLD, liver cirrhosis, and HCC, as well as diabetes mellitus, hypertension, hyperlipidemia, chronic renal insufficiency, and HIV infection. For the diagnosis of viral hepatitis, all patients underwent HBsAg and HCV antibody testing.^16^ Nucleic acid tests for HCV RNA and HBV DNA were performed for all patients who were positive for HCV antibodies and HBsAg, respectively. Patients with active HCC were classified based on radiographic evidence of an HCC lesion on a 4‐phase multidetector computed tomography scan or dynamic contrast‐enhanced magnetic resonance imaging. The imaging had to demonstrate arterial hypervascularity and venous or delayed phase washout, and it was performed within 12 months of the patient's enrollment. The diagnosis of NAFLD was based on liver echotexture (bright liver and hepatorenal echo contrast), deep attenuation (diaphragm visibility), and vessel blurring (intrahepatic vessel visibility) on ultrasonography after excluding secondary causes of hepatic fat accumulation such as significant alcohol consumption, long‐term use of a steatogenic medication and HBV or HCV infection.^17^ Liver cirrhosis was diagnosed through imaging studies including ultrasonography, computed tomography, and magnetic resonance imaging showing the presence of cirrhotic changes in the liver. Decompensation was defined as a total bilirubin level greater than 2 mg/dL or a prothrombin time prolongation of more than 2 s. We excluded patients who met the following criteria: (1) age less than 20 years, and (2) having HIV infection, chronic renal deficiency, or receiving maintenance hemodialysis therapy, which are known to be associated with suboptimal immune responses to COVID‐19 vaccination. These exclusion criteria were applied to ensure that the participants had a sufficient immune response to the third booster dose of BNT162b2 or mRNA‐1273, which was the focus of the study.^18^, ^19^ The definition of chronic liver disease (HBV infection, HCV infection, NAFLD, liver cirrhosis, and HCC) was reviewed and reported by experienced hepatologists. The control group consisted of individuals who did not have HIV or chronic liver disease as defined by chronic HBV or HCV infection, NAFLD, liver cirrhosis, or HCC. These individuals were included in the study to provide a comparison group for the vaccinated participants with chronic liver disease who received a third booster dose of BNT162b2 or mRNA‐1273. The inclusion criteria of the control group were designed to ensure that the control group did not have any preexisting liver diseases or conditions that may have affected their immune response to the COVID‐19 vaccine.

### 2.3 Immunogenicity and types of vaccine booster

We evaluated the effects of homologous and heterologous vaccine boosters on anti‐S IgG levels in individuals with and without chronic liver disease. Three vaccine regimens were used: AZAZBNT, AZAZM, and MMM. (1) AZAZBNT: first dose: AZD1222; second dose: AZD1222; third dose: BNT162b2; (2) AZAZM: first dose: AZD1222; second dose: AZD1222; third dose: mRNA‐1273; (3) MMM: first dose: mRNA‐1273; second dose: mRNA‐1273; third dose: mRNA‐1273. SARS‐CoV‐2‐IgG spike antibody levels were measured twice during the study, at visit 1 (mean 1.5 ± 0.7 months; range: 1–3 months) and visit 2 (mean 5.0 ± 0.5 months; range: 4–6 months) after the third booster of BNT162b2 or mRNA‐1273 vaccine, using Abbott's SARS‐CoV‐2 IgG II assay. The results were reported as concentrations in AU/mL, which are highly correlated with the World Health Organization International Standard (binding antibody unit, BAU) (Abbott: BAU/mL = 0.142 × AU/mL). A positive reactive result was considered using a cut‐off of 17.8 BAU/mL or more. Additionally, a threshold of 4160 AU/mL was considered as a surrogate marker for serum neutralizing activity, which we defined as significant antibody activity.^20^ The anti‐SARS‐CoV‐2 nucleocapsid antibody was analyzed using a chemiluminescent microparticle immunoassay (Abbott, SARS‐CoV‐2 IgG II, cutoff ≥1.4 S/C).

### 2.4 Statistical analysis

The Mann–Whitney *U* or Kruskal–Wallis test was used to compare demographic characteristics, vaccine types, and chronic liver disease. Fisher's exact or the *χ* ^2^ test was used to compare the positive and negative significant antibody activity groups. The Wilcoxon signed‐rank test was used to compare anti‐S IgG levels between visit 1 and visit 2 in the patients with chronic liver disease. Multiple logistic regression analysis was used to examine the correlation between chronic liver disease and significant antibody activity.

A sensitive analysis was also conducted to examine the relationship between significant antibody activity and chronic liver disease, with individuals without chronic liver disease serving as the control group. The control group consisted of 132 individuals without HBV, HCV, NAFLD, liver cirrhosis, and HCC. Patients with HBV (*n* = 323), HCV (*n* = 194), NAFLD (*n* = 90), liver cirrhosis (*n* = 65), and HCC (*n* = 48) were compared to the control group to investigate the relationship between significant antibody activity and each of these conditions separately. We conducted all analyses using SPSS (version 22; IBM, Armonk, NY, USA). The significance level was set at *p* < 0.05.

## 3 RESULTS

### 3.1 Demographic characteristics and anti‐S IgG levels

A total of 722 subjects were included in the study, of whom 85 (11.8%), 308 (42.7%), and 329 (45.6%) received three doses of AZAZBNT, AZAZM, and MMM vaccine boosters, respectively (Table 1). Significantly more of the older individuals (MMM: 64.3 ± 11.3 vs. AZAZM: 54.0 ± 13.2 and AZAZBNT: 50.6 ± 11.3 years, *p* < 0.001), those with diabetes mellitus (MMM: 22.8% vs. AZAZM: 12.7% and AZAZBNT: 9.4%, *p* < 0.001), hypertension (MMM: 40.4% vs. AZAZM: 23.7% and AZAZBNT: 15.3%, *p* < 0.001), HCV infection (MMM: 34.3% vs. AZAZM: 22.1% and AZAZBNT: 15.3%, *p* < 0.001), and liver cirrhosis (MMM: 12.5% vs. AZAZM: 6.2% and AZAZBNT: 5.9%, *p* = 0.012) received the MMM vaccine booster regimen. Significantly more of the patients with liver cirrhosis and liver decompensation (MMM: 9.8% vs. AZAZM: 10.5% and AZAZBNT: 60.0%, *p* = 0.008) received the AZAZBNT vaccine booster regimen (Table 1).

**TABLE 1:** Baseline characteristics of all participants (*n* = 722).

Item	AZAZBNT (*n* = 85)	AZAZM (*n* = 308)	MMM (*n* = 329)	*p*
Continuous variable, mean ± SD (range)				
Age (years)	50.6 ± 11.3	54.0 ± 13.2	64.3 ± 11.3	<0.001
BMI (kg/m^2^)	24.6 ± 4.5	24.8 ± 4.4	24.9 ± 3.7	0.225
Category variable, *n* (%)				
Gender				0.316
Female	45 (52.9)	167 (54.2)	159 (48.3)	
Male	40 (47.1)	141 (45.8)	170 (51.7)	
Diabetes mellitus				<0.001
No	77 (90.6)	269 (87.3)	254 (77.2)	
Yes	8 (9.4)	39 (12.7)	75 (22.8)	
Hypertension				<0.001
No	72 (84.7)	235 (76.3)	196 (59.6)	
Yes	13 (15.3)	73 (23.7)	133 (40.4)	
Hyperlipidemia				0.061
No	76 (89.4)	266 (86.4)	266 (80.9)	
Yes	9 (10.6)	42 (13.6)	63 (19.1)	
Chronic liver disease				
HBsAg				0.682
Negative	44 (51.8)	175 (56.8)	180 (54.7)	
Positive	41 (48.2)	133 (43.2)	149 (45.3)	
HBV DNA				0.174
Negative	25 (61.0)	78 (58.6)	103 (69.1)	
Positive	16 (39.0)	55 (41.4)	46 (30.9)	
HBV NUCs treatment				0.492
No	16 (39.0)	62 (46.6)	60 (40.3)	
Yes	25 (61.0)	71 (53.4)	89 (59.7)	
HCV				<0.001
Negative	72 (84.7)	240 (77.9)	216 (65.7)	
Positive	13 (15.3)	68 (22.1)	113 (34.3)	
HCV RNA				0.767
Negative	12 (92.3)	65 (95.6)	109 (96.5)	
Positive	1 (7.7)	3 (4.4)	4 (3.5)	
HCV DAA treatment				0.537
No	11 (84.6)	57 (83.8)	101 (89.4)	
Yes	2 (15.4)	11 (16.2)	12 (10.6)	
NAFLD				0.043
No	80 (94.1)	260 (84.4)	291 (88.4)	
Yes	5 (5.9)	48 (15.6)	38 (11.6)	
NASH				0.208
No	2 (40.0)	17 (36.2)	20 (55.6)	
Yes	3 (60.0)	30 (63.8)	16 (44.4)	
Liver cirrhosis				0.012
No	80 (94.1)	289 (93.8)	288 (87.5)	
Yes	5 (5.9)	19 (6.2)	41 (12.5)	
Liver decompensation				0.008
No	2 (40.0)	17 (89.5)	37 (90.2)	
Yes	3 (60.0)	2 (10.5)	4 (9.8)	
HCC				0.296
No	81 (95.3)	291 (94.5)	302 (91.8)	
Yes	4 (4.7)	17 (5.5)	27 (8.2)	
Active HCC				0.278
No	1 (25.0)	8 (47.1)	17 (63.0)	
Yes	3 (75.0)	9 (52.9)	10 (37.0)	
HCC with target/immunotherapy				0.597
No	4 (100)	14 (82.4)	24 (88.9)	
Yes	0 (0)	3 (17.6)	3 (11.1)	

*Note*: The first, second, and third booster vaccines, listed in order, were (1) AZAZBNT, (2) AZAZM, and (3) MMM. In this list, AZ, BNT, and M corresponded to the vaccines AZD1222, BNT162b2, and mRNA‐1273, respectively.

Abbreviations: HCC, hepatocellular carcinoma; NAFLD, non‐alcoholic fatty liver disease.

### 3.2 Comparison of anti‐S IgG levels between homologous and heterologous vaccines

**FIGURE 1:**
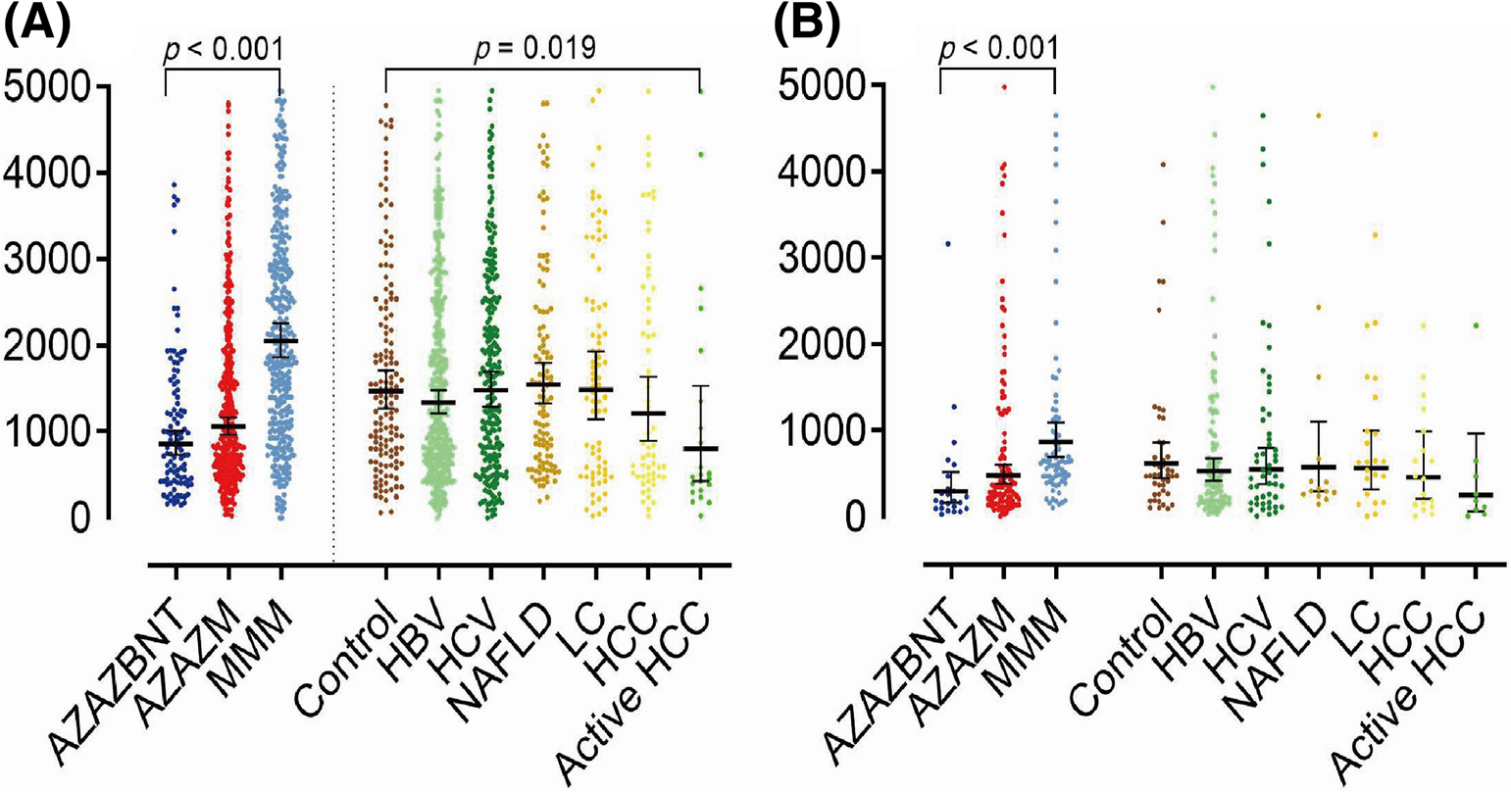
Distribution of levels of anti‐S‐IgG (geometric mean) (A) MMM booster had significantly highest anti‐S‐IgG levels in visit 1 (*p* < 0.001) and visit 2 (*p* < 0.001). Active HCC had lower anti‐S IgG levels than control (*p* = 0.019). (B) No significant difference observed in anti‐S IgG levels between the chronic liver disease patients and the control group. Visit 1: 1.5 ± 0.7 months and visit 2: 5.0 ± 0.5 months after the third vaccine booster. The first, second, and third booster vaccines, listed in order, were (1) AZAZBNT, (2) AZAZM, and (3) MMM. In this list, AZ, BNT, and M corresponded to the vaccines AZD1222, BNT162b2, and mRNA‐1273, respectively.

The levels of nucleocapsid antibodies in all participants were below 1.4 S/C, indicating that none of them had previously been infected with SARS‐CoV‐2 (Table S1). The participants who received MMM boosters had significantly higher levels of anti‐S IgG (geometric mean, BAU/mL) than those who received AZAZBNT and AZAZM boosters (MMM: 2150.1; AZAZM: 1044.7 and AZAZBNT: 881.7; *p* < 0.001) at visit 1 and (MMM: 868.5; AZAZM: 482.9 and AZAZBNT: 311.9; *p* < 0.001) at visit 2 (Figure 1A,B, Table S1). Males had significantly higher levels of anti‐S IgG (geometric mean, BAU/mL) than females at visit 1 (1651.2 vs. 1236.0; *p* < 0.001), but no significant difference was found at visit 2 (Table S2).

**FIGURE 2:**
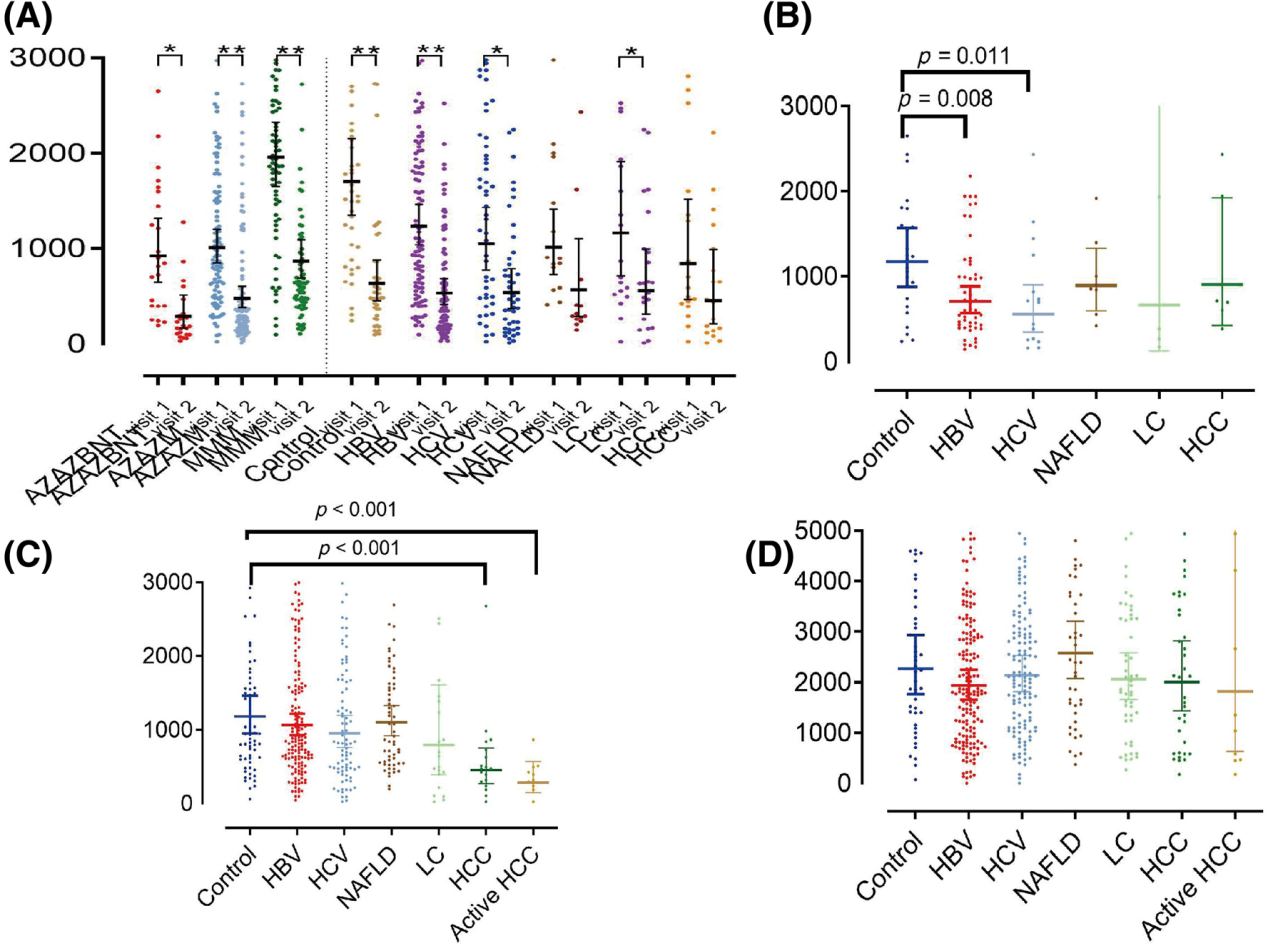
(A) Wilcoxon sign rank test compare anti‐S IgG levels (geometric mean) between visit 1 and visit 2. *p < 0.05; **p < 0.01. Compared levels of anti‐S IgG between patients with chronic liver disease and control group by vaccine types. (B) AZAZBNT, (C) AZAZM, and (D) MMM.

Using the Wilcoxon signed‐rank test for pairwise comparisons, we found that the levels of anti‐S IgG (geometric mean, BAU/mL) in the patients who received AZAZBNT (311.9 vs. 975.5, *p* = 0.011), AZAZM (521.9 vs. 1035.4, *p* = 0.001), and MMM (919.0 vs. 1976.6, *p* < 0.001) boosters, and those with HBV infection (567.8 vs. 1251.6, *p* < 0.001), liver cirrhosis (676.9 vs. 1266.4, *p* = 0.042), and the controls (636.1 vs. 1696.0, *p* < 0.001) at visit 2 were significantly lower than those at visit 1 (Figure 2A, Table S3).

At visit 1, the patients with chronic liver disease who received AZAZBNT vaccine boosters had a significantly lower level of anti‐S IgG (geometric mean, BAU/mL) compared to those without chronic liver disease (738.7 vs. 1235.7; *p* = 0.027) (Table S4). In addition, the male patients had significantly higher anti‐S‐IgG levels (geometric mean, BAU/mL) compared to the female patients after receiving AZAZBNT (1122.1 vs. 719.6; *p* = 0.027), AZAZM (1298.7 vs. 866.4; *p* = 0.001), and MMM (2549.5 vs. 2100.0; *p* = 0.009) vaccine boosters (Table S4).

### 3.3 Significant antibody activity in the patients with chronic liver disease after the third vaccine booster at visit 1

More of the patients with significant antibody activity were male (52.0% vs. 48.0% females, *p* < 0.001) and received MMM vaccine boosters (51.5% MMM vs. 38.9% AZAZM and 9.5% AZAZBNT, *p* < 0.001). They also had lower rates of diabetes mellitus (15.0% with diabetes vs. 85.0% without diabetes, *p* = 0.005), liver cirrhosis with decompensation (0.7% decompensation vs. 8.0% non‐decompensation and 91.3% non‐liver cirrhosis, *p* = 0.015), and active HCC (1.5% active HCC vs. 3.7% non‐active HCC and 94.7% non‐HCC, *p* < 0.001) (Table 2). Multiple logistic regression analysis revealed that those without HCC (odds ratio [OR] = 6.39, 95% CI 2.09–19.50, *p* = 0.001) and without active HCC (OR = 8.81, 95% CI 1.73–44.81, *p* = 0.009) had a significantly increased odds of having significant antibody activity compared to those with active HCC. In addition, receiving MMM vaccine boosters (OR = 10.67, 95% CI 5.20–21.91, *p* < 0.001) significantly increased the odds of having significant antibody activity compared with AZAZBNT vaccine boosters (Table 2).

**TABLE 2:** Variables associated with significant antibody activity to COVID‐19 vaccination within visit 1 (*n* = 722).

Item	No	Yes	*p*	Univariate			Multivariate		
				OR	95% CI	*p*	OR	95% CI	*p*
Age (years)	59.5 ± 12.2	58.0 ± 13.6	0.615	0.99	0.99 (0.98–1.01)	0.229	0.97	(0.95–0.99)	0.003
BMI (kg/m^2^)	24.9 ± 4.7	24.8 ± 3.9	0.660	0.99	0.99 (0.95–1.04)	0.708			
Category variable *n* (%)									
Sex			<0.001			<0.001			0.002
Female	89 (66.4)	282 (48.0)		ref			ref		
Male	45 (33.6)	306 (52.0)		2.15	2.15 (1.45–3.18)		2.06	(1.31–3.25)	
Hyperlipidemia			0.511			0.408			
No	116 (86.6)	492 (83.7)		ref					
Yes	18 (13.4)	96 (16.3)		1.26	1.26 (0.73–2.16)				
Hypertension			0.835			0.778			
No	92 (68.7)	411 (69.9)		ref					
Yes	42 (31.3)	177 (30.1)		0.94	0.94 (0.63–1.42)				
Diabetes mellitus			0.005			0.004			0.010
No	100 (74.6)	500 (85.0)		ref			ref		
Yes	34 (25.4)	88 (15.0)		0.52	0.52 (0.33–0.81)		0.46	(0.26–0.83)	
Chronic liver disease									
HBV			0.925						
Non‐NUCs	24 (17.9)	114 (19.4)		ref					
NUCs	35 (26.1)	150 (25.5)		0.90	0.90 (0.51–1.60)	0.725			
Non‐HBV	75 (56.0)	324 (55.1)		0.91	0.91 (0.55–1.51)	0.714			
HCV			0.776						
Non‐DAA	31 (23.1)	138 (23.5)		ref					
DAA	6 (4.5)	19 (3.2)		0.71	0.71 (0.26–1.93)	0.503			
Non‐HCV	97 (72.4)	431 (73.3)		1.00	1.00 (0.64–1.56)	0.993			
NAFLD			0.232						
Abnormal liver function	12 (9.0)	37 (6.3)		ref					
Normal liver function	4 (3.0)	35 (6.0)		2.84	2.84 (0.84–9.64)	0.094			
Non‐NAFLD	118 (88.1)	516 (87.8)		1.42	1.42 (0.72–2.80)	0.315			
Liver cirrhosis			0.015						
Decompensation	5 (3.7)	4 (0.7)		ref					
Non‐decompensation	9 (6.7)	47 (8.0)		6.53	6.53 (1.46–29.13)	0.014			
Non‐liver cirrhosis	120 (89.6)	537 (91.3)		5.59	5.59 (1.48–21.14)	0.011			
HCC			<0.001						
Active HCC	13 (9.7)	9 (1.5)		ref			ref		
Non‐active HCC	4 (3.0)	22 (3.7)		7.94	7.94 (2.03–31.04)	0.003	8.81	(1.73–44.81)	0.009
Non‐HCC	117 (87.3)	557 (94.7)		6.88	6.88 (2.87–16.46)	<0.001	6.39	(2.09–19.50)	0.001
Vaccine types			<0.001						
AZAZBNT	29 (21.6)	56 (9.5)		ref			ref		
AZAZM	79 (59.0)	229 (38.9)		1.50	1.50 (0.90–2.52)	0.123	1.71	(0.96–3.04)	0.070
MMM	26 (19.4)	303 (51.5)		6.04	6.04 (3.31–11.01)	<0.001	10.67	(5.20–21.91)	<0.001

*Note*: Visit 1: 1.5 ± 0.7 months.

### 3.4 Comparison of anti‐S IgG levels between the patients with chronic liver disease and the control group

The average ages (in years) of the patients with chronic liver diseases, including HBV (58.1 ± 11, *p* < 0.001), HCV (65.7 ± 9.3, *p* < 0.001), NAFLD (57.6 ± 13.1, *p* < 0.001), liver cirrhosis (66.4 ± 11.0, *p* < 0.001), and HCC (68.8 ± 9.7, *p* < 0.001) were higher compared to the control group (49.6 ± 16.3, *p* < 0.001). Furthermore, the average body mass index (in kg/m^2^) values of the patients with HBV (24.8 ± 3.9, *p* < 0.001), HCV (24.4 ± 3.9, *p* = 0.002), NAFLD (28.1 ± 4.5, *p* < 0.001), liver cirrhosis (25.6 ± 3.5, *p* < 0.001), and HCC (25.2 ± 3.7, *p* = 0.007) were higher compared to the control group (23.4 ± 3.5). The proportion of males was significantly lower in the patients with HBV (41.2%, *p* < 0.001), NAFLD (37.8%, *p* = 0.001), liver cirrhosis (46.2%, *p* = 0.048), and HCC (27.1%, *p* < 0.001) compared to the control group (61.4%). Furthermore, the prevalence of diabetes mellitus and hypertension was significantly higher in those with HBV (16.1%, *p* = 0.036; 29.7%, *p* = 0.048), HCV (19.1%, *p* = 0.007; 37.6%, *p* = 0.001), NAFLD (27.8%, *p* < 0.001; 42.2%, *p* = 0.001), liver cirrhosis (36.9%, *p* < 0.001; 47.7%, *p* < 0.001), and HCC (39.6%, *p* < 0.001; 52.1%, *p* < 0.001) compared to the control group (Table 3).

**TABLE 3:** Comparison of demographic characteristics and levels of anti‐S IgG between patients with chronic liver disease patients and control group.

Items	HBV (*n* = 323)	HCV (*n* = 194)	NAFLD (*n* = 90)	LC (*n* = 65)	HCC (*n* = 48)	Active HCC (*n* 22)	Control (*n* = 132)	*p* ^a^	*p* ^b^	*p* ^c^	*p* ^d^	*p* ^e^	*p* ^f^
Continuous variable, mean ± SD (range)													
Age(years)	58.1 ± 11.0	65.7 ± 9.3	57.6 ± 13.1	66.4 ± 11.0	68.8 ± 9.7	68.1 ± 9.9	49.6 ± 16.3	**<0.001**	**<0.001**	**<0.001**	**<0.001**	**<0.001**	**<0.001**
BMI (kg/m^2^)	24.8 ± 3.9	24.4 ± 3.9	28.1 ± 4.5	25.6 ± 3.5	25.2 ± 3.7	25.4 ± 4.0	23.4 ± 3.5	**<0.001**	**0.002**	**<0.001**	**<0.001**	**0.007**	**0.027**
Anti‐S IgG levels (BAU/mL), geometric mean													
Visit 1	1367.4	1516.0	1476.9	1604.3	1105.4	761.6	1498.2	0.406	0.525	0.850	0.630	0.094	**0.019**
Visit 2	567.8	620.8	614.7	676.9	495.9	315.7	636.1	0.472	0.828	0.435	0.618	0.640	0.174
Category variable, *n* (%)													
Gender								**<0.001**	1.000	**0.001**	**0.048**	**<0.001**	**0.011**
Female	190 (58.8)	74 (38.1)	56 (62.2)	35 (53.8)	35 (72.9)	15 (68.2)	51 (38.6)						
Male	133 (41.2)	120 (61.9)	34 (37.8)	30 (46.2)	13 (27.1)	7 (31.8)	81 (61.4)						
DM								**0.036**	**0.007**	**<0.001**	**<0.001**	**<0.001**	**<0.001**
No	271 (83.9)	157 (80.9)	65 (72.2)	41 (63.1)	29 (60.4)	12 (54.5)	121 (91.7)						
Yes	52 (16.1)	37 (19.1)	25 (27.8)	24 (36.9)	19 (39.6)	10 (45.5)	11 (8.3)						
Hypertension								**0.048**	**0.001**	**0.001**	**<0.001**	**<0.001**	**<0.001**
No	227 (70.3)	121 (62.4)	52 (57.8)	34 (52.3)	23 (47.9)	8 (36.4)	105 (79.5)						
Yes	96 (29.7)	73 (37.6)	38 (42.2)	31 (47.7)	25 (52.1)	14 (63.6)	27 (20.5)						
Hyperlipidemia								0.393	1.000	**0.031**	0.818	1.000	1.000
No	269 (83.3)	170 (87.6)	68 (75.6)	58 (89.2)	42 (87.5)	20 (90.9)	115 (87.1)						
Yes	54 (16.7)	24 (12.4)	22 (24.4)	7 (10.8)	6 (12.5)	2 (9.1)	17 (12.9)						
Significant antibody activity, anti‐S‐IgG levels (BAU/mL)													
Visit 1								0.341	0.298	0.459	0.227	**0.003**	**<0.001**
≤4160	59 (18.3)	37 (19.1)	17 (18.9)	14 (21.5)	17 (35.4)	13 (59.1)	19 (14.4)						
>4160	264 (81.7)	157 (80.9)	73 (81.1)	51 (78.5)	31 (64.6)	9 (40.9)	113 (85.6)						
Visit 2								1.000	0.296	1.000	0.188	0.778	0.720
≤4160	63 (57.3)	26 (50.0)	10 (66.7)	10 (41.7)	10 (50.0)	6 (66.7)	26 (56.5)						
>4160	47 (42.7)	26 (50.0)	5 (33.3)	14 (58.3)	10 (50.0)	3 (33.3)	20 (43.5)						

*Note*: The control group consisted of 132 individuals who were free from HBV, HCV, NAFLD, liver cirrhosis, and HCC. Active HCC defined as the presence of a lesion on a 4‐phase multidetector computed tomography scan or dynamic contrast‐enhanced MR imaging, exhibiting arterial hypervascularity and washout in the venous or delayed phase within 1 year. Visit 1: 1.5 ± 0.7 months and visit 2: 5.0 ± 0.5 months after the third vaccine booster. Bold values: *p* < 0.05.

^a^ HBV versus control.

^b^ HCV versus control.

^c^ NAFLD versus control.

^d^ LC versus control.

^e^ HCC versus control.

^f^ Active HCC versus control.

We also found that the patients with active HCC had a significantly lower level of anti‐S IgG (geometric mean, BAU/mL) than the controls (active HCC: 761.6 vs. controls: 1498.2, *p* = 0.019) at visit 1 (Figure 1A, Table 3). However, there was no significant difference in anti‐S IgG level between the controls and patients with chronic liver disease at visit 2 (Figure 1B, Table 3).

In the AZAZBNT vaccine booster group, we observed that the anti‐S IgG levels (geometric mean, BAU/mL) in the patients with HBV (728.5; *p* = 0.008) and HCV (562.6; *p* = 0.011) were significantly lower compared to the control group (1175.3) (Figure 2B, Table S5). Furthermore, in the AZAZM vaccine booster group, the anti‐S IgG level (geometric mean, BAU/mL) in the patients with HCC was significantly lower than that in the control group (453.1 vs. 1180.8, *p* < 0.001) (Figure 2C, Table S5).

### 3.5 Sensitive analysis of significant antibody activity among the patients with chronic liver disease and control group at visit 1

We investigated the relationship between significant antibody activity and liver cirrhosis (Table S6), HCC (Table S7), HBV infection (Table S8), HCV infection (Table S9), and NAFLD (Table S10). The analysis showed that the control group (OR = 10.67, 95% CI 5.20–21.91, *p* < 0.001) and non‐active HCC (OR = 10.67, 95% CI 5.20–21.91, *p* < 0.001) significantly increased the likelihood of having significant antibody activity compared to active HCC (Table 4).

**TABLE 4:** Compared significant antibody activity to the third dose of the SARS‐CoV‐2 vaccine between patients with chronic liver disease and control group.

Item	Multivariate		
	OR	95% CI	*p*
HBV			0.314
HBV	ref		
Control	1.39	(0.73–2.66)	
HCV			0.654
HCV	ref		
Control	1.20	(0.55–2.62)	
NAFLD			0.718
NAFLD	ref		
Control	1.19	(0.46–3.06)	
Liver cirrhosis			
Decompensation	ref		
Non‐decompensation	5.46	(0.97–30.85)	0.055
Control	3.12	(0.70–13.91)	0.136
HCC			
Active HCC	ref		
Non‐active HCC	12.92	(1.88–88.93)	0.009
Control	10.16	(1.29–80.33)	0.028

## 4 DISCUSSION

This study is the first to explore the anti‐SARS‐CoV‐2 spike antibody response in chronic liver disease patients following the third homologous or heterologous vaccine booster. Our results showed that receiving three doses of homologous mRNA‐1273 vaccine booster significantly increased the rate of antibody activity compared to receiving a heterologous vaccine booster regimen with BNT162b2 as the third dose in this patient population. However, we observed that active HCC patients had a significantly lower immunogenic response compared to control subjects after receiving three doses of vaccination.

Previous studies have demonstrated that heterologous mRNA boosting may provide better protection against incident infection in individuals initially vaccinated with an adenoviral vector vaccine,^10^, ^21^, ^22^ but no significant difference in the incidence of SARS‐CoV‐2 infection in those who received primary mRNA vaccination.^23^ In mouse models, heterologous boosting of CoronaVac with one of three different vaccines has been shown to result in comparable or higher neutralizing levels than homologous boosting, due to the induction of cellular immune response dominated by cytotoxic T cells and Th1+ CD4 T cells.^24^ These findings suggest that different vaccine platforms may lead to significant heterogeneity in study conclusions. Our results demonstrated that a homologous mRNA‐1273 three‐dose booster regimen provided the highest rate of significant antibody activity in chronic liver disease patients. Therefore, further research is needed to clarify the impact of homologous or heterologous vaccine boosters on this patient population.

A previous study of 74 HCC patients who received two doses of inactivated SARS‐CoV‐2 vaccines reported a positive neutralizing antibody rate of 60.8% (median: 13.5 AU/mL).^25^ In our study, we found a higher significant antibody activity rate (anti‐S IgG > 4160 AU/mL) of 69.2% in the HCC patients, but the rate for patients with active HCC was not reported in the previous study. We also found that the patients with active HCC had a lower rate of significant antibody activity (41.7%), with only 10% in the AZAZM group, and 63.6% in the MMM group. The EASL recommends that patients with chronic liver disease should receive SARS‐CoV‐2 vaccination, with priority given to patients with advanced cirrhosis, liver decompensation, and hepatobiliary cancer. However, they do not provide a suggested regimen for vaccines.^14^ In contrast, the AASLD Expert Panel Consensus Statement recommends that mRNA COVID‐19 vaccines should be administered to immunosuppressed patients according to their standard dose and schedule, as they are expected to have a favorable efficacy and safety profile.^26^ Our findings are compatible with the AASLD recommendations, as the mRNA‐1273 vaccine booster had a higher rate of positive serum neutralizing activity in the patients with chronic liver disease.

In our study, we observed a significant decrease in the level of anti‐S‐IgG at visit 2 (which took place 3–6 months after visit 1) compared to the level observed at visit 1. Specifically, the rates of positive significant antibody activity in the patients with chronic liver disease and active HCC decreased to 45.9% and 30.0%, respectively, at visit 2. This rapid decline in anti‐S‐IgG level raises concerns about the need for a fourth vaccine booster. Retrospective cohort studies have revealed that a fourth dose of BNT162b2 is immunogenic for individuals aged 60 years or older, and that both BNT162b2 and mRNA‐1273 are immunogenic for healthy young healthcare workers.^27^ However, the safety and efficacy of a fourth vaccine dose for patients with chronic liver disease remain unknown.

Patients with chronic liver disease may have a diminished immune response to SARS‐CoV‐2 vaccine boosters, which may be due to the intricate involvement of the liver in both innate and adaptive immunity. The compromised functionality of immune cells, such as macrophages and dendritic cells, influenced by chronic liver disease can impact antigen presentation and cytokine production.^28^ Altered expressions of pattern recognition receptors such as Toll‐like receptors and dysregulation of the complement system in the liver may contribute to a weakened immune response, potentially affecting the efficacy of SARS‐CoV‐2 vaccine boosters.^29^ Furthermore, adaptive immune dysfunction in these patients, involving defects in B and T cell functions, may lead to poor responses to booster vaccinations, with impaired T‐cell and antibody responses observed in patients with cirrhosis after COVID‐19 vaccination. Altered B cell activity and dysregulation of the Th1/Th2 lymphocyte ratio, along with abnormalities in the complement system, could collectively compromise the effectiveness of adaptive immune responses to SARS‐CoV‐2 boosters in individuals with chronic liver disease.^30^ Previous research has shown that individuals with chronic liver disease who have cirrhosis may have reduced or inadequate responses to COVID‐19 vaccines.^28^ This is likely due to immune dysfunction associated with advanced cirrhosis, which is characterized by a range of immune system alterations seen in end‐stage liver disease. These immune system changes may contribute to the increased risk and severity of COVID‐19 in patients with cirrhosis, as well as a potential decrease in the effectiveness of the vaccine.^31^ A recent analysis of national registries found that cirrhosis was associated with a lower humoral response to the COVID‐19 vaccine, as evidenced by lower levels of anti‐S IgG antibodies 14 days after the second vaccine dose.^32^ Specifically, the humoral response was considered to be lower if anti‐S IgG levels were below 418.95 nM.^32^ In our study, we observed that the patients with liver cirrhosis and decompensation had a significantly lower rate of antibody activity. However, the association between liver cirrhosis and antibody activity was not significant in multivariate logistic regression analysis. While the vaccine type used in both studies (mRNA‐1273 and BNT162b2, except for ChAdOx1 nCoV‐19) was similar, differences in ethnicity, case numbers, and definition of chronic liver disease between the studies may explain the discrepancies in the results. For instance, the proportion of patients with cirrhosis was much higher in their study than in ours (61.8% vs. 12.5%), although the number of HCC patients in their study was relatively low (*n* = 15). In addition, their study did not find a significant difference in anti‐S IgG levels between patients with and without HCC. Differences in the selection criteria for chronic liver disease patients may also have contributed to the differences in results between the studies. Furthermore, our study did not consider a humoral response cut‐off level of 418.95 nM as indicative of significant antibody activity.

Recent recommendations from the AASLD strongly advocate vaccination and booster shots for individuals with chronic liver disease and liver transplant recipients.^13^ Emphasizing the importance of a three‐dose mRNA COVID‐19 vaccine regimen for immunosuppressed patients, the guidelines stress timely administration, especially as liver disease severity progresses with time. Despite the proven value of current vaccines in addressing the challenge of COVID‐19 in chronic liver disease patients, concerns persist about their effectiveness, safety, and durability, particularly considering diverse disease etiologies and levels of immunosuppression. In this study, we compared homologous and heterologous booster regimens with the aim of guiding booster recommendations. Our results suggest that homologous boosters may be preferential for chronic liver disease patients, and we also identified subgroups of patients, such as those with active HCC, who may have a reduced immune response to the third booster. We also correlated antibody response levels with clinical outcomes, aiding clinicians in tailoring vaccination strategies for specific patient populations within the broader chronic liver disease category.

A previous study indicated that a two‐dose mRNA‐1273 regimen was more effective in preventing medically attended COVID‐19 across various settings than a two‐dose BNT162b2 regimen,^33^ particularly among individuals with a history of blood/stem cell or solid organ transplant or those undergoing active immunosuppressive therapy. Another large‐scale study using data from 38 World Health Organization COVID‐19 databases recommended mRNA boosters to supplement primary vaccine courses.^34^ The study found comparable efficacy between heterologous and homologous three‐dose regimens in preventing COVID‐19 infections, but caution was advised in interpreting the results for immunocompromised patients due to limited data.^34^ In addition, a study focusing on individuals who did not seroconvert after two mRNA vaccinations revealed that homologous mRNA‐1273 booster vaccinations were more effective in inducing seroconversion compared to heterologous vaccinations. These findings underscore the importance of booster vaccinations, especially in immunocompromised patients, with superior efficacy observed with homologous mRNA‐1273 regimens.

Prioritizing MMM booster regimens, which showed significantly higher anti‐S IgG levels, particularly in the patients with chronic liver disease in this study, could enhance vaccine response. Healthcare providers need to be mindful that individuals with chronic liver diseases, especially those with HBV or HCV, may have lower anti‐S IgG levels after the AZAZBNT booster. Considering alternative booster options such as MMM may be preferable for this population. Personalized follow‐up strategies, including more frequent monitoring or additional booster doses, may be warranted due to the varied responses among different patient groups. Healthcare providers should educate patients about potential differences in vaccine responses based on their underlying liver condition and discuss individualized vaccination plans, incorporating clinical judgment and evolving scientific evidence into decision‐making.

One of the strengths of our study is its prospective, multi‐hospital design, which allowed us to assess the immunogenic response to COVID‐19 vaccination in chronic liver disease patients. In addition, we were able to exclude subjects with previous SARS‐CoV‐2 infection from the study, which increased the validity of our findings. However, we must also acknowledge some limitations of our study. First, the sample size of certain subgroups of chronic liver disease patients, such as those with liver cirrhosis or HCC, was relatively small. Second, the IgG threshold of 4160 AU/mL that we used might be considered conservative, although it has been correlated with neutralization capacity in many clinical studies.^20^ Third, selection bias was possible, specifically with regards to age and body mass index. These biases could have arisen due to the way we recruited our participants or due to differences in the baseline characteristics of the chronic liver disease and control groups.

## 5 CONCLUSIONS

In this study, the patients with active HCC had lower anti‐S‐IgG levels and less significant antibody activity response after the third vaccine booster. Homologous mRNA‐1273 vaccines significantly increased the odds of significant antibody activity in the chronic liver disease patients. These findings underscore the importance of booster vaccinations, especially in immunocompromised patients, with superior efficacy observed with the homologous mRNA‐1273 regimen.

## CONFLICT OF INTEREST STATEMENT

The authors declare no conflict of interest.

## Supporting information


**Data S1.** Supporting Information.
