# Supplementary material for: Third vaccine boosters and anti‐S‐IgG levels: A comparison of homologous and heterologous responses and poor immunogenicity in hepatocellular carcinoma
Source: Kaohsiung J Med Sci. 2024 Feb 16;40(5):477–88. doi: 10.1002/kjm2.12812 (PMC11895633; doi:10.1002/kjm2.12812)
Supplement: Supplementary file 1 — Data S1. Supporting Information. [file KJM2-40-477-s001.docx]

**Comparison of Anti-S-IgG Levels Following a Third Dose of Homologous or Heterologous Vaccine Boosters, and Poor Immunogenic Response in Patients with Hepatocellular Carcinoma**

**Supplementary** **materials**

**Table S1** Distribution of anti-S IgG titers following the third booster vaccine at two separate visits, as well as the levels of nucleocapsid antibodies

**Table S2** Compared the levels of anti-S IgG between different demographic characteristics at visit 1 and visit 2 following the third booster vaccine (n= 722)

**Table S3** Wilcoxon Signed Ranks test compared paired anti-S IgG levels between visit 1 and visit 2 following the third booster vaccine

**Table S4** Difference of anti-S IgG levels by homologues or heterologous vaccine booster within visit 1 (n= 722)

**Table S5** Compared levels of anti-S IgG between patients with chronic liver disease and control group by vaccine type within visit 1

**Table S6** Comparing the significant antibody response to the third SARS-COV-2 vaccine between liver cirrhosis patients and control group

**Table S7** Comparing the significant antibody response to the third SARS-COV-2 vaccine between HCC patients and control group

**Table S8** Comparing the significant antibody response to the third SARS-COV-2 vaccine between HBV patients and control group

**Table S9** Comparing the significant antibody response to the third SARS-COV-2 vaccine between HCV patients and control group

**Table S10** Comparing the significant antibody response to the third SARS-COV-2 vaccine between NAFLD patients and control group

Table S1 Distribution of anti-S IgG titers following the third booster vaccine at two separate visits, as well as the levels of nucleocapsid antibodies

| Item | Mean | SD | GM | Min. | 5th | 10th | 25th | Median | 75th | 90th | 95th | Max. | p |
| --- | --- | --- | --- | --- | --- | --- | --- | --- | --- | --- | --- | --- | --- |
| **Visit 1 (n=722)** | . | . | . | . | . | . | . | . | . | . | . | . | <0.001 |
| Anti-S IgG (BAU/mL) | | | | | | | | | | | | |  |
| AZAZBNT (n=85) | 1309.6 | 1386.0 | 881.7 | 163.9 | 199.2 | 256.3 | 458.0 | 898.5 | 1674.7 | 2919.5 | 3819.9 | 7671.6 |  |
| AZAZM (n=308) | 1701.3 | 2424.6 | 1044.7 | 25.5 | 192.3 | 308.9 | 571.7 | 1036.6 | 2045.1 | 3517.7 | 4796.0 | 27239.8 |  |
| MMM (n=329) | 3082.2 | 2914.0 | 2150.1 | 0.6 | 434.2 | 694.6 | 1345.2 | 2506.8 | 3962.2 | 5927.5 | 7562.3 | 33597.0 |  |
| Anti-S IgG (AU/mL) | | | | | | | | | | | | | <0.001 |
| AZAZBNT | 9222.2 | 9760.8 | 6209.1 | 1154.2 | 1402.5 | 1804.8 | 3225.4 | 6327.5 | 11793.3 | 20560.1 | 26900.4 | 54025.4 |  |
| AZAZM | 11981.3 | 17074.4 | 7357.3 | 179.6 | 1354.5 | 2175.1 | 4026.1 | 7300.0 | 14401.8 | 24772.5 | 33774.8 | 191829.6 |  |
| MMM | 21705.9 | 20521.1 | 10020.3 | 4.2 | 3057.8 | 4891.6 | 9472.9 | 17653.5 | 27902.5 | 41743.0 | 53255.6 | 236598.6 |  |
| **Visit 2 (n=199)** | | | | | | | | | | | | |  |
| Anti-S IgG (BAU/mL) | | | | | | | | | | | | | <0.001 |
| AZAZBNT (n=25) | 1040.6 | 2239.2 | 311.9 | 35.7 | 43.6 | 68.5 | 113.8 | 232.6 | 632.3 | 4583.0 | 8593.3 | 9398.8 |  |
| AZAZM (n=102) | 1088.0 | 1657.2 | 482.9 | 13.2 | 87.4 | 117.8 | 202.1 | 385.4 | 1251.3 | 3418.1 | 5017.7 | 10036.0 |  |
| MMM (n=72) | 1649.1 | 2461.2 | 868.5 | 108.1 | 173.7 | 235.7 | 468.8 | 651.4 | 1409.6 | 4564.9 | 7939.6 | 14322.3 |  |
| Anti-S IgG (AU/mL) | | | | | | | | | | | | | <0.001 |
| AZAZBNT | 7328.0 | 15769.3 | 2196.7 | 251.4 | 307.2 | 482.5 | 801.4 | 1638.0 | 4452.8 | 32274.9 | 60516.0 | 66188.7 |  |
| AZAZM | 7662.3 | 11670.1 | 3401.0 | 93.0 | 615.4 | 829.6 | 1423.2 | 2714.1 | 8812.0 | 24071.3 | 35335.7 | 70676.1 |  |
| MMM | 11613.7 | 17332.5 | 6116.0 | 761.3 | 1223.2 | 1660.0 | 3301.4 | 4587.3 | 9926.8 | 32147.0 | 55912.8 | 100861.3 |  |
| **Anti-SARS-CoV-2 nucleocapsid antibody (S/C)** | | | | | | | | | | | | |  |
| AZAZBNT | 0 | 0.07 | 0 | 0 | 0 | 0 | 0 | 0 | 0 | 0.02 | 0.06 | 0.60 |  |
| AZAZM | 0 | 0.02 | 0 | 0 | 0 | 0 | 0 | 0 | 0 | 0.02 | 0.03 | 0.20 |  |
| MMM | 0 | 0.03 | 0 | 0 | 0 | 0 | 0 | 0 | 0 | 0.02 | 0.03 | 0.50 |  |

The first, second, and third booster vaccines, listed in order, were (1) AZAZBNT, (2) AZAZM, and (3) MMM. In this list, AZ, BNT, and M corresponded to the vaccines AZD1222, BNT162b2, and mRNA-1273, respectively. GM: geometric mean. Visit 1: 1.5 ± 0.7 months and visit 2: 5.0 ± 0.5 months after the third vaccine booster.

Table S2 Compared the levels of anti-S IgG between different demographic characteristics at visit 1 and visit 2 following the third booster vaccine (n= 722)

| Items | Anti-S IgG titers (BAU/mL), geometric mean | | | |  | Case numbers, n (%). | |
| --- | --- | --- | --- | --- | --- | --- | --- |
|  | visit 1 | p | visit 2 | p |  | Visit1 | visit 2 |
| Gender | . | **<0.001** | . | 0.093 |  | . | . |
| Female | 1236.0 | . | 519.2 | . |  | 371(51.4) | 98(49.2) |
| Male | 1651.2 | . | 691.2 | . |  | 351(48.6) | 101(50.8) |
| Diabetes mellitus | . | 0.704 | . | 0.504 |  | . | . |
| No | 1447.9 | . | 591.7 | . |  | 600(83.1) | 168(84.4) |
| Yes | 1305.9 | . | 649.3 | . |  | 122(16.9) | 31(15.6) |
| Hypertension | . | 0.252 | . | 0.286 |  | . | . |
| No | 1377.6 | . | 587.1 | . |  | 503(69.7) | 136(68.3) |
| Yes | 1532.7 | . | 671.7 | . |  | 219(30.3) | 63(31.7) |
| Hyperlipidemia | . | 0.617 | . | 0.437 |  | . | . |
| No | 1409.9 | . | 569.5 | . |  | 608(84.2) | 166(83.4) |
| Yes | 1494.1 | . | 589.9 | . |  | 114(15.8) | 33(16.6) |
| Chronic liver disease | . | 0.629 | . | 0.942 |  | . | . |
| No | 1497.7 | . | 598.5 | . |  | 144(19.9) | 47(23.6) |
| Yes | 1404.8 | . | 600.9 | . |  | 578(80.1) | 152(76.4) |

Visit 1: 1.5 ± 0.7 months and visit 2: 5.0 ± 0.5 months after the third vaccine booster.

Table S3 Wilcoxon Signed Ranks test compared paired anti-S IgG levels between visit 1 and visit 2 following the third booster vaccine

| Items | N | visit 1 | visit 2 | p |
| --- | --- | --- | --- | --- |
| Anti-S IgG titers (BAU/mL), geometric mean | | | | |
| AZAZBNT | 25 | 975.5 | 311.9 | **0.011** |
| AZAZM | 102 | 1035.4 | 521.9 | **0.001** |
| MMM | 72 | 1976.6 | 919.0 | **<0.001** |
| Chronic liver disease |  |  |  |  |
| HBV | 99 | 1251.6 | 567.8 | **<0.001** |
| HCV | 46 | 1095.4 | 620.8 | 0.052 |
| NAFLD | 13 | 1002.6 | 614.7 | 0.807 |
| Liver cirrhosis | 22 | 1266.4 | 676.9 | **0.042** |
| HCC | 18 | 798.7 | 495.9 | 0.215 |
| Control | 42 | 1696.0 | 636.1 | **<0.001** |

The control group were individuals free from HBV, HCV, NAFLD, liver cirrhosis, and HCC.

Table S4 Difference of anti-S IgG levels by homologues or heterologous vaccine booster within visit 1 (n= 722)

| Anti-S IgG titers (BAU/mL)  geometric mean | AZAZBNT  (n= 85) | p | AZAZM  (n= 308) | p | MMM  (n= 329) | p |
| --- | --- | --- | --- | --- | --- | --- |
| Gender | . | **0.027** | . | **0.001** | . | **0.009** |
| Female | 719.6 | . | 866.4 | . | 2100.0 | . |
| Male | 1122.1 | . | 1298.7 | . | 2549.5 | . |
| BMI (kg/m^2^) | . | 0.657 | . | 0.052 | . | 0.762 |
| BMI<24 | 913.7 | . | 942.9 | . | 2506.8 | . |
| BMI≥24 | 859.6 | . | 1163.3 | . | 2269.6 | . |
| Diabetes mellitus | . | 0.338 | . | **0.008** | . | 0.492 |
| No | 898.5 | . | 1106.3 | . | 2390.2 | . |
| Yes | 445.2 | . | 675.4 | . | 2164.8 | . |
| Hypertension | . | 0.261 | . | 0.865 | . | 0.477 |
| No | 898.5 | . | 1058.3 | . | 2311.7 | . |
| Yes | 859.6 | . | 1020.4 | . | 2274.6 | . |
| Hyperlipidemia | . | 0.438 | . | 0.782 | . | 0.620 |
| No | 928.9 | . | 1076.85 | . | 2432 | . |
| Yes | 845.3 | . | 1040.7 | . | 2132.5 | . |
| Chronic liver disease | . | **0.027** | . | 0.307 | . | 0.645 |
| No | 1235.7 | . | 1160.9 | . | 2506.8 | . |
| Yes | 738.7 | . | 1044.9 | . | 2248.0 | . |

Table S5 Compared levels of anti-S IgG between patients with chronic liver disease and control group by vaccine type within visit 1

| Items | HBV | HCV | NAFLD | LC | HCC | Control | P^a^ | P^b^ | P^c^ | P^d^ | P^e^ |
| --- | --- | --- | --- | --- | --- | --- | --- | --- | --- | --- | --- |
| **Anti-S IgG levels (BAU/mL), geometric mean** | | | | | | | | | | | |
| AZAZBNT | 728.5 | 562.6 | 896.1 | 670.3 | 907.9 | 1175.3 | **0.008** | **0.011** | 0.207 | 0.448 | 0.439 |
| AZAZM | 1064.7 | 960.2 | 1105.2 | 796.2 | 453.1 | 1180.8 | 0.503 | 0.326 | 0.656 | 0.158 | **<0.001** |
| MMM | 1937.3 | 2143.8 | 2578.5 | 2069.2 | 2011.6 | 2274.9 | 0.289 | 0.741 | 0.552 | 0.420 | 0.399 |
| **Case numbers** |  |  |  |  |  |  |  |  |  |  |  |
| AZAZBNT (n= 85) | 41 | 13 | 5 | 5 | 4 | 22 |  |  |  |  |  |
| AZAZM (n= 308) | 133 | 68 | 48 | 19 | 17 | 63 |  |  |  |  |  |
| MMM (n= 329) | 149 | 113 | 37 | 41 | 27 | 47 |  |  |  |  |  |

^a^HBV vs. control; ^b^HCV vs. control; ^c^NAFLD vs. control; ^d^LC vs. control; ^e^HCC vs. control.

Table S6 Comparing the significant antibody response to the third SARS-COV-2 vaccine between liver cirrhosis patients and control group

| Items | Univariate | | |  | Multivariate | | |
| --- | --- | --- | --- | --- | --- | --- | --- |
|  | OR | 95% CI | p |  | OR | 95% CI | p |
| Age | 0.97 | (0.95,1.00) | 0.029 |  | 0.96 | (0.93,1.00) | 0.064 |
| Sex (male vs. female) | 3.13 | (1.42,6.88) | 0.005 |  | 3.62 | (1.39,9.48) | 0.009 |
| BMI | 1.01 | (0.91,1.12) | 0.877 |  | 1.16 | (0.99,1.37) | 0.075 |
| Hyperlipidemia | 1.47 | (0.41,5.24) | 0.554 |  | 5.08 | (0.89,29.04) | 0.068 |
| Hypertension | 0.58 | (0.27,1.27) | 0.172 |  | 0.68 | (0.21,2.21) | 0.526 |
| Diabetes mellitus | 0.41 | (0.18,0.97) | 0.043 |  | 0.31 | (0.08,1.16) | 0.082 |
| Vaccine types | 1.94 | (1.15,3.28) | 0.014 |  | 2.83 | (1.46,5.47) | 0.002 |
| HBV (yes vs. no) | 1.49 | (0.49,4.58) | 0.484 |  | 2.12 | (0.44,10.20) | 0.348 |
| HCV (yes vs. no) | 0.67 | (0.26,1.71) | 0.399 |  | 2.43 | (0.37,16.07) | 0.358 |
| HCC (yes vs. no) | 0.37 | (0.14,1.01) | 0.051 |  | 0.52 | (0.08,3.21) | 0.479 |
| LC (Control vs. LC) | 1.63 | (0.76,3.51) | 0.209 |  | 1.77 | (0.39,8.06) | 0.460 |

Table S7 Comparing the significant antibody response to the third SARS-COV-2 vaccine between HCC patients and control group

| Items | Univariate | | |  | Multivariate | | |
| --- | --- | --- | --- | --- | --- | --- | --- |
|  | OR | 95% CI | p |  | OR | 95% CI | p |
| Age | 0.97 | (0.94,0.99) | 0.006 |  | 0.97 | (0.93,1.01) | 0.176 |
| Sex (male vs. female) | 2.65 | (1.23,5.70) | 0.013 |  | 2.81 | (1.03,7.67) | 0.043 |
| BMI | 1.00 | (0.90,1.11) | 0.993 |  | 1.22 | (1.03,1.44) | 0.022 |
| Hyperlipidemia | 1.77 | (0.50,6.34) | 0.377 |  | 6.45 | (0.98,42.42) | 0.053 |
| Hypertension | 0.31 | (0.15,0.66) | 0.002 |  | 0.37 | (0.11,1.26) | 0.111 |
| Diabetes mellitus | 0.29 | (0.12,0.67) | 0.004 |  | 0.28 | (0.06,1.26) | 0.096 |
| Vaccine types | 1.48 | (0.88,2.49) | 0.137 |  | 2.29 | (1.16,4.52) | 0.017 |
| HBV (yes vs. no) | 0.78 | (0.26,2.28) | 0.643 |  | 2.56 | (0.34,19.25) | 0.362 |
| HCV (yes vs. no) | 0.33 | (0.13,0.80) | 0.014 |  | 2.51 | (0.32,19.57) | 0.381 |
| LC (yes vs. no) | 0.48 | (0.18,1.29) | 0.145 |  | 0.49 | (0.06,4.04) | 0.505 |
| HCC |  |  |  |  |  |  |  |
| Active HCC | ref | . | . |  | ref | . | . |
| Non-active HCC | 7.94 | (2.03,31.04) | 0.003 |  | 12.92 | (1.88,88.93) | 0.009 |
| Control | 8.59 | (3.23,22.87) | <0.001 |  | 10.16 | (1.29,80.33) | 0.028 |

Table S8 Comparing the significant antibody response to the third SARS-COV-2 vaccine between HBV patients and control group

| Items | Univariate | | |  | Multivariate | | |
| --- | --- | --- | --- | --- | --- | --- | --- |
|  | OR | 95% CI | p |  | OR | 95% CI | p |
| Age | 0.99 | (0.97,1.01) | 0.214 |  | 0.97 | (0.95,1.00) | 0.025 |
| Sex (male vs. female) | 1.99 | (1.19,3.32) | 0.008 |  | 1.95 | (1.09,3.50) | 0.024 |
| BMI | 1.02 | (0.96,1.09) | 0.517 |  | 1.09 | (1.00,1.18) | 0.052 |
| Hyperlipidemia | 1.15 | (0.58,2.31) | 0.688 |  | 1.46 | (0.66,3.24) | 0.347 |
| Hypertension | 0.65 | (0.38,1.09) | 0.099 |  | 0.49 | (0.25,0.96) | 0.037 |
| Diabetes mellitus | 0.61 | (0.32,1.16) | 0.133 |  | 0.60 | (0.27,1.32) | 0.200 |
| Vaccine types | 2.55 | (1.78,3.64) | <0.001 |  | 3.48 | (2.29,5.30) | <0.001 |
| HCV (yes vs. no) | 5.00 | (0.67,37.61) | 0.118 |  | 4.84 | (0.53,43.88) | 0.161 |
| LC (yes vs. no) | 1.48 | (0.51,4.36) | 0.472 |  | 1.20 | (0.34,4.19) | 0.780 |
| HCC (yes vs. no) | 0.65 | (0.23,1.82) | 0.410 |  | 0.56 | (0.16,1.94) | 0.359 |
| HBV (control vs. HBV) | 1.33 | (0.76,2.33) | 0.321 |  | 1.39 | (0.73,2.66) | 0.314 |

Table S9 Comparing the significant antibody response to the third SARS-COV-2 vaccine between HCV patients and control group

| Items | Univariate | | |  | Multivariate | | |
| --- | --- | --- | --- | --- | --- | --- | --- |
|  | OR | 95% CI | p |  | OR | 95% CI | p |
| Age | 0.98 | (0.96,1.00) | 0.089 |  | 0.97 | (0.94,1.00) | 0.024 |
| Sex (male vs. female) | 2.13 | (1.19,3.80) | 0.011 |  | 2.03 | (1.04,3.96) | 0.039 |
| BMI | 1.04 | (0.96,1.12) | 0.400 |  | 1.08 | (0.97,1.21) | 0.153 |
| Hyperlipidemia | 2.89 | (0.86,9.73) | 0.086 |  | 2.58 | (0.63,10.53) | 0.186 |
| Hypertension | 1.13 | (0.60,2.13) | 0.708 |  | 1.00 | (0.43,2.32) | 0.999 |
| Diabetes mellitus | 0.56 | (0.27,1.17) | 0.123 |  | 0.53 | (0.20,1.44) | 0.215 |
| Vaccine types | 2.54 | (1.66,3.88) | <0.001 |  | 3.16 | (1.89,5.30) | <0.001 |
| LC (yes vs. no) | 0.71 | (0.29,1.74) | 0.46 |  | 1.13 | (0.28,4.49) | 0.866 |
| HCC (yes vs. no) | 0.29 | (0.12,0.68) | 0.004 |  | 0.39 | (0.10,1.53) | 0.178 |
| HBV (yes vs. no) | 5.12 | (0.68,38.74) | 0.114 |  | 4.90 | (0.55,43.67) | 0.155 |
| HCV (control vs. HCV) | 1.40 | (0.77,2.56) | 0.273 |  | 1.20 | (0.55,2.62) | 0.654 |

Table S10 Comparing the significant antibody response to the third SARS-COV-2 vaccine between NAFLD patients and control group

| Items | Univariate | | |  | Multivariate | | |
| --- | --- | --- | --- | --- | --- | --- | --- |
|  | OR | 95% CI | p |  | OR | 95% CI | p |
| Age | 0.99 | (0.96,1.01) | 0.227 |  | 0.98 | (0.95,1.01) | 0.192 |
| Sex (male vs. female) | 1.87 | (0.90,3.87) | 0.093 |  | 1.98 | (0.86,4.53) | 0.107 |
| BMI | 0.97 | (0.90,1.04) | 0.381 |  | 1.02 | (0.92,1.13) | 0.758 |
| Hyperlipidemia | 0.70 | (0.29,1.68) | 0.424 |  | 1.11 | (0.40,3.13) | 0.841 |
| Hypertension | 0.80 | (0.37,1.71) | 0.560 |  | 1.16 | (0.45,2.97) | 0.757 |
| Diabetes mellitus | 0.35 | (0.16,0.81) | 0.013 |  | 0.26 | (0.09,0.74) | 0.012 |
| Vaccine types | 1.86 | (1.08,3.19) | 0.025 |  | 2.80 | (1.46,5.37) | 0.002 |
| HBV (yes vs. no) | 2.20 | (0.28,17.59) | 0.457 |  | 2.15 | (0.22,20.91) | 0.510 |
| NAFLD (control vs. NAFLD) | 1.39 | (0.68,2.84) | 0.374 |  | 1.19 | (0.46,3.06) | 0.718 |
